# Water Colour Shapes Diving Beetle (Coleoptera: Dytiscidae) Assemblages in Urban Ponds

**DOI:** 10.3390/insects15050308

**Published:** 2024-04-25

**Authors:** Wenfei Liao

**Affiliations:** 1 School of Life Science, University of Electronic Science and Technology of China, No. 4, Section 2, North Jianshe Road, Chengdu 610054, China; wenfei.liao@helsinki.fi; 2 Ecosystems and Environment Research Programme, University of Helsinki, P.O. Box 65, FI-00014 Helsinki, Finland; 3 Department of Geosciences and Geography, Faculty of Science, University of Helsinki, P.O. Box 64, FI-00014 Helsinki, Finland; 4 Helsinki Institute of Urban and Regional Studies (Urbaria), FI-00100 Helsinki, Finland

**Keywords:** aquatic insect, biodiversity conservation, city, ecosystem service, impermeable surface, impervious surface, lentic habitat, macroinvertebrate, people’s perception, wetland

## Abstract

**Simple Summary:**

Water colour is an important physicochemical property of water that affects aquatic communities. Land-use change has led to water colour darkening in lotic habitats, such as streams and rivers. Here, I evaluate whether urban land-use change affects water colour in urban ponds, a type of lentic habitats, and how diving beetles (Dytiscidae) respond to the water colour gradient in the Helsinki Metropolitan Area, Finland. The results show that urbanisation may not drive water darkening in urban ponds, possibly because urban ponds are often not connected to stormwater pipes. Diving beetles responded to the increasing pond water colour differently in the presence or absence of fish. Diving beetle species richness and abundance significantly increased along the water colour gradient in ponds with fish, but not in ponds without fish. Some species, such as the great diving beetle (*Dytiscus marginalis*), appear tolerant to brown water, whereas some species, such as the cherrystone beetle (*Hyphydrus ovatus*), prefer clear water. This study highlights that not all species benefit from increasing water colour. It is important to provide ponds with a gradient of water colour from clear to brown water in the landscape to meet the habitat requirements of different species for urban biodiversity conservation.

**Abstract:**

Dramatic land-use changes in urban landscapes can drive water colour darkening by washing compounds, such as organic matter and iron, from terrestrial ecosystems into urban blue space, consequentially affecting aquatic communities. Here, I studied how pond water colour changes along an urban gradient and how diving beetles (Dytiscidae) respond to the water colour gradient in 11 ponds with fish and 15 ponds without fish in the Helsinki Metropolitan Area, Finland. I found that the pond water colour exhibited a non-significant decreasing pattern along the urban gradient, indicating that urbanisation may not necessarily drive brownification in urban ponds. Dytiscid species richness and abundance exhibited significant positive correlations with increasing water colour in ponds with fish but no significant correlation in ponds without fish. Some species, such as *Agabus* spp. and *Dytiscus* spp., appeared tolerant to highly coloured water, whereas some species, such as *Hyphydrus ovatus* and *Hygrotus* spp., tended to occur in clear water, indicating that brown water may provide dytiscids with prey refuges, but some species are intolerant to brown water. The study highlights the importance of urban pondscape heterogeneity to meet the needs of aquatic invertebrates that prefer different water colours and for the multifunctioning of urban ponds.

## 1. Introduction

The dramatic land-use change in urban areas, transforming natural and semi-natural habitats into impermeable surfaces, has induced hydrological changes in cities [1,2,3]. Urbanisation, together with recent climate change, has led to increasing surface runoff, which changes water quality in urban blue space [4], such as increasing concentrations of organic matter and iron and changing water colour towards yellow–brown hues [5,6,7]. Such changes in water quality have consequential effects on ecosystem services, such as drinking water supply and biodiversity conservation [8,9,10].

Water colour is an important physicochemical property of water that affects aquatic ecosystems via mechanics, such as altering light availability and heat penetration [11,12,13]. It affects primary producers by regulating photosynthesis, nutrient availability, and competition [13,14,15]. Water colour is also a determinant of aquatic macroinvertebrate assemblages and species compositions in both lentic and lotic habitats [16,17,18,19,20,21] and affects ecosystem functioning by altering the variance in macroinvertebrate functional composition [22]. Thus, changes in water colour can alter aquatic communities, which, consequentially, affects ecosystem functioning. 

The change in surface water colour towards yellow–brown hues, known as brownification or browning, is a global phenomenon in freshwater ecosystems, especially in the Northern Hemisphere [23,24,25]. Water browning has been driven by many factors, such as land-use change and climate change [26,27,28,29]. Land-use change, such as transforming agricultural lands into forests, increases concentrations of dissolved organic carbon (DOC) and iron (Fe) in aquatic ecosystems, resulting in increasing water colour [28,29,30,31]. In urban contexts, concentrations of DOC and Fe in streams and rivers are often found to exhibit increasing patterns along rural–urban gradients [5,6,32,33,34,35,36]. As water colour affects both the ecological and social values of urban wetlands [21,22,37], it is important to understand the patterns of water colour in urban landscapes. However, most urban studies related to water colour were conducted in lotic ecosystems, whereas little research has investigated whether urbanisation drives water colour change in lentic habitats, such as urban ponds, and how the water colour gradient affects aquatic biodiversity in such habitats. 

Macroinvertebrates, as an important link between primary producers and predators at high trophic positions, such as fish and waterbirds, provide many ecosystem services, including nutrient cycling and educational values [38,39,40,41]. Among aquatic macroinvertebrates, diving beetles (Coleoptera: Dytiscidae) have become model taxa of ecology and evolution [42,43] and have been utilised as indicator taxa for aquatic macroinvertebrate conservation [44,45,46,47,48,49,50,51]. In urban ponds, dytiscid diversity is affected by both landscape and local conditions, such as landscape connectivity and the presence or absence of fish predators [52,53,54]. Investigating the effects of water colour on dytiscids in an urban landscape can deepen our understanding of how aquatic biodiversity changes in the browning aquatic world and bring insights into aquatic conservation.

In this research, I utilise diving beetles (Coleoptera: Dytiscidae) as the focal taxa to investigate how water colour affects aquatic macroinvertebrate diversity in urban ponds. Here, I aim to answer three main research questions: (1) Does pond water colour change along the urban gradient? (2) How does water colour affect dytiscid assemblages in urban ponds? (3) Does the effect of water colour on dytiscids differ in the presence or absence of fish? 

## 2. Materials and Methods

### 2.1. Study Taxa

Diving beetles (Dytiscidae) have adapted to living in aquatic environments. Dytiscid larvae are predators, feeding on other aquatic invertebrates, such as mosquito larvae, and even vertebrates, such as small fish and tadpoles [55]. Dytiscid larvae usually climb out from water for pupation [56]. Most dytiscid species have aquatic adults, while a few dytiscid species are considered terrestrial [57,58]. The adults of some species, such as *Dytiscus* spp., overwinter in water, while some species, such as *Ilybius* spp., overwinter on land [59,60]. Most dytiscids are early colonisers, because they can disperse actively via flight while utilising anemochory, i.e., utilising wind for passive dispersal, for long-distance movement; therefore, they occur in many different types of wetlands, including urban ponds [60,61]. Water beetles have been utilized as indicator taxa of freshwater biodiversity, because their diversity is positively correlated with the diversity of other aquatic invertebrates [46,47].

### 2.2. Data Collection

I surveyed dytiscids and water colour in 26 urban ponds at 11 sites in the Helsinki Metropolitan Area (60.17° N, 24.94° E), Finland. Eleven ponds had fish and fifteen ponds were fishless. The water pH of the ponds varied from 5.88 to 8.96 (mean ± SD = 7.03 ± 0.52). To collect dytiscids, I set five activity traps in the smallest pond and fifteen traps in the pond with the longest perimeter; the shortest pond perimeter was 59 m and the longest was 559 m (210 ± 144 m). Each activity trap consisted of a 1 L glass jar and a plastic funnel with 10 cm at the large end and 2.5 cm at the narrow end. I operated the traps without bait and placed them horizontally in water for 48 h [61,62]. Most sites were sampled for four rounds from May to August 2021. The dytiscid specimens were preserved in 70% ethanol until identification with a microscope in the laboratory. All specimens were identified to the species level according to the taxonomic keys [59,63]. The nomenclature of the identified species followed the world catalogue of Dytiscidae 2022 [64].

To collect water colour data, I used 200 mL water bottles to collect water samples for laboratory analysis. One water sample was collected for each wetland every round and kept in cold (4 °C) until analysis. To obtain water colour data, I utilised the Lovibond^®^ comparator for visual comparison of the water samples to the distilled water with coloured glass disks, which had been calibrated to correspond to Hazen’s platinum–cobalt scale. When the water colour of some ponds exceeded the scale of the coloured glass disks, 1:2 dilution or 1:4 dilution was performed to fit the scale. The unit of water colour produced was mgPt/L.

To obtain the land-use data, I utilised the open dataset of the Helsinki Metropolitan Area land cover in 2020 [65] with the software QGIS version 3.36 [66] and calculated the percentage of impermeable surfaces in a 500 m buffer from the shore of a pond as an indicator of how urbanised a pond surrounding was. A 500 m buffer was chosen to standardise the quantification of urbanisation while minimising the effects of the sea. The extent of urbanisation in the pond surroundings varied from 18.71% to 60.97% (mean ± SD = 33.84 ± 10.97%).

### 2.3. Generalised Linear Mixed Models

I applied generalised linear mixed models (GLMMs) to investigate how pond water colour changed along the urban gradient. Before data analysis, the percentage of impermeable surfaces was standardized with Z-score transformation, to improve the numerical optimisation process of GLMMs [67]. In data exploration, I fitted GLMMs with a Poisson distribution, with the water colour as the response variables; the urban gradient as the fixed effects; and the ponds, the sites, and the sampling months as random effects. However, Poisson models could not cope with the overdispersion in the residuals. Therefore, I applied GLMMs with negative binomial distributions with the same components [67]. 

In total, I collected 663 activity traps during the four sampling months, with 202 traps in May, 176 traps in June, 146 traps in July, and 116 traps in August. Among all the traps, 475 (71.6%) did not have any dytiscid. I applied GLMMs to investigate how dytiscid species richness and abundance changed along the water colour gradient. In data exploration, I noticed the interaction between water colour and the presence or absence of fish affected dytiscid species richness and abundance; therefore, I included water colour, the presence or absence of fish, and their interaction as fixed effects in the initial GLMM models with Poisson distributions. The water colour data were standardized with Z-score transformation before data analysis to improve the numerical optimisation process [67]. As the data are nested, I included the sites, the ponds, and the sampling months as random effects in the initial models to avoid pseudo-replication; the random effects also deal with the unmeasured environmental factors in the models [67,68]. As GLMMs with a Poisson distribution could not deal with the overdispersion in the abundance data, I applied GLMMs with a negative binomial distribution to investigate how dytiscid abundance changed along the water colour gradient. The full models are described in Appendix A. 

I selected models manually with a backward selection. I selected the optimal model with the lowest AIC (Akaike information criterion) values [67]. In cross validation, I plotted the residuals of the optimal models against each covariate to ensure that there was no pattern in the residuals. I simulated 10,000 datasets with the optimal models to ensure that the optimal models were able to cope with the high percentage of zeros in the dataset of dytiscid species richness and abundance and it was unnecessary to run zero-inflated models [69]. 

### 2.4. Non-Metric Multidimensional Scaling 

I applied non-metric multidimensional scaling (NMDS) to analyse how dytiscid assemblages responded to a gradient of water colour, as NMDS is a useful method to represent interrelationships among a set of observations [70]. I pooled the observations per month per pond, yielding 81 observations in total. I obtained 55 samples with dytiscids, 19 samples in May, 17 samples in June, 10 samples in July, and 9 samples in August. The other 26 samples had no dytiscid beetles. Thus, only 55 dytiscid assemblage samples were included in the NMDS analysis.

I applied NMDS analysis with Bray–Curtis dissimilarity to investigate the similarities between water beetle assemblages, as Bray–Curtis dissimilarity is a good choice for detecting gradients [71]. Although much of the previous literature utilised ‘0.20’ as a cutoff for the acceptable stress of an NMDS [72,73,74], stress is positively correlated with increasing sample size [75]. To find the reliable minimal number of axes, in data exploration, I conducted NMDS ordination on 1000 independent permutations of the original water beetle assemblage dataset with different numbers of axes (k); the minimal k was 3 in this case and the stress was 0.098, which can be considered as good configuration and indicates no need to increase the number of axes [76]. I fitted the environmental factors to the NMDS analysis to investigate how water colour affected dytiscid assemblage compositions in the presence or absence of fish. Other environmental factors included the urban gradient and the sampling months, because they are known to affect dytiscid assemblages [54,61], but NMDS does not include random effects as GLMMs.

All data analyses were conducted with the R software, version 4.2.2 [77]. I used the “glmmTMB” package [78] to build GLMMs and the “vegan” package to apply NMDS analysis [79]. 

## 3. Results

### 3.1. Water Colour Had Little Change along the Urban Gradient

**Figure 1:**
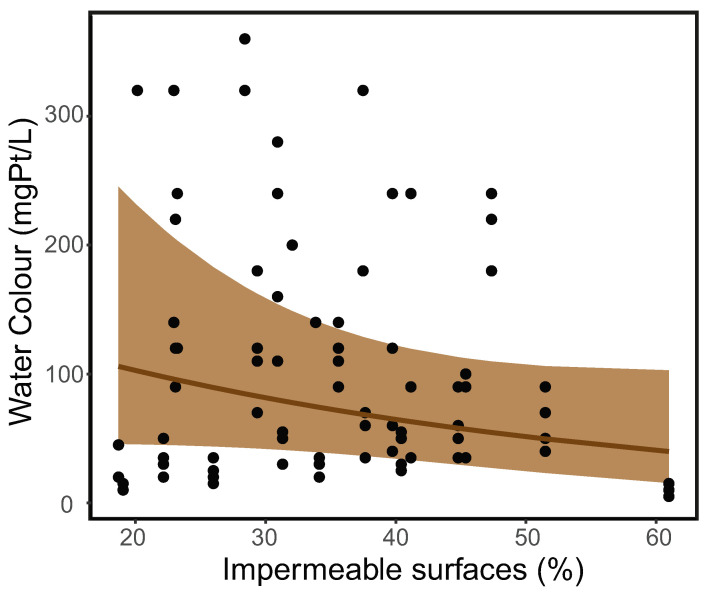
Water colour exhibits a non-significant decrease along the urban gradient in the study ponds. The brown ribbon stands for the 95% confidence interval.

The water colour in the study ponds varied from 5 to 360 mgPt/L. The water colour in ponds with fish varied from 10 to 220 mgPt/L (mean ± SD = 55 ± 40 mgPt/L), whereas the water colour in ponds without fish varied from 5 to 360 mgPt/L (150 ± 100 mgPt/L). The negative binomial GLMM result showed that water colour decreased along the urban gradient of 18.71% to 60.97% impermeable surfaces in the pond surroundings but without statistical significance (estimated parameter = −0.25; Z-value = −1.51; *p*-value = 0.130, Figure 1). The variance of sites as random effects was 0.90^2^. The variance of ponds as random effects was 0.45^2^. The variance of months as random effects was 0.25^2^.

### 3.2. Effects of Water Colour on Dytiscid Species Richness and Abundance 

In total, I recorded 645 dytiscid specimens of 44 species under 18 genera during the sampling in 2021, with 143 specimens of 29 species in May, 198 specimens of 30 species in June, 111 specimens of 26 species in July, and 193 specimens of 27 species in August. The species are listed in Appendix B.

**Figure 2:**
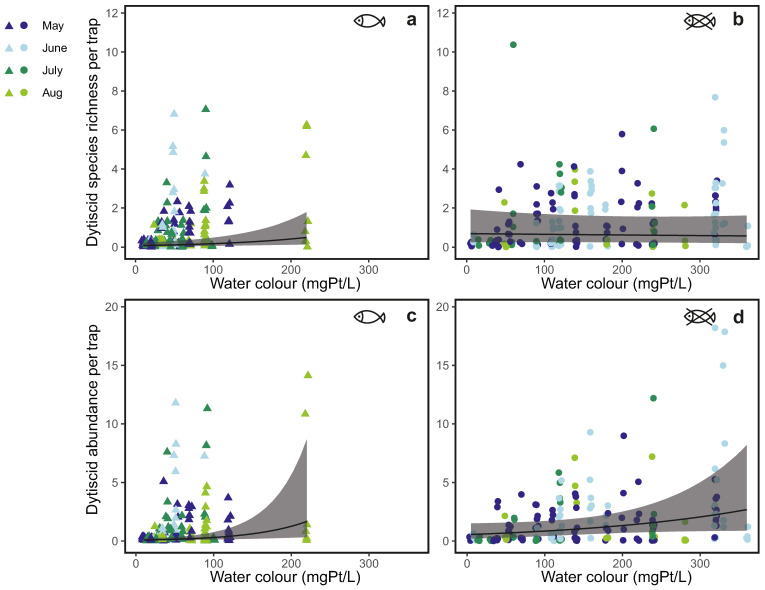
Dytiscid species richness and abundance along the water colour gradient in ponds with fish (**a**,**c**) and in ponds without fish (**b**,**d**). The grey ribbons stand for 95% confidence intervals.

The optimal Poisson GLMM results showed that dytiscid species richness was affected by the interaction between water colour and the presence or absence of fish (*p*-value = 0.002). Dytiscid species richness increased significantly along the water colour gradient in *ponds with fish* (Z-value = 3.13, *p*-value = 0.002; Table 1; Figure 2a) but was not affected by water colour in *ponds without fish* (Z-value = −0.40, *p*-value = 0. 693; Figure 2b). Dytiscid species richness was significantly higher in ponds without fish than in ponds with fish (Z-value = 2.53, *p*-value = 0.011).

The optimal GLMM with a negative binomial distribution showed that dytiscid abundance was also affected by the interaction between water colour and the presence or absence of fish (*p*-value < 0.001). Dytiscid abundance increased along the water colour gradient significantly in *ponds with fish* (Z-value = 4.72, *p*-value < 0.001; Table 2; Figure 2c) but without statistical significance in *ponds without fish* (Z-value = −0.74, *p*-value = 0.458; Figure 2d). Dytiscids were significantly more abundant in ponds without fish than in ponds with fish (Z-value = 2.92, *p*-value = 003).

### 3.3. Dytiscid Assemblages Changed along the Water Colour Gradient

**Figure 3:**
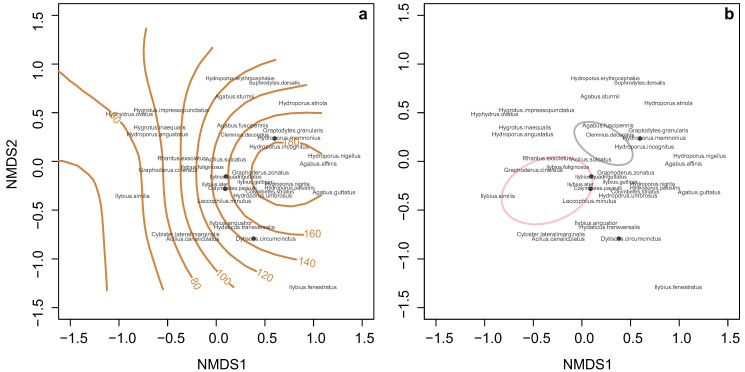
NMDS plots of dytiscid assemblages: (**a**) along the water colour gradient; (**b**) in ponds with (pink) and without fish (grey). The pink and grey circles stand for 95% confidence intervals.

The NMDS results showed that water colour was a determinant of dytiscid assemblages (r^2^ = 0.20, *p*-value = 0.004; Figure 3a). Dytiscid assemblages were significantly different in ponds with fish and ponds without fish (r^2^ = 0.13, *p*-value = 0.002; Figure 3b). Dytiscid assemblages were also affected by the urban gradient (r^2^ = 0.26, *p*-value = 0.001; Figure S6a) and the sampling months (r^2^ = 0.14, *p*-value = 0.008; Figure S6b). The stress of the NMDS was 0.097.

## 4. Discussion

In this study, I investigated whether urbanisation drove water colour change in urban ponds and how dytiscid assemblages change along a gradient of water colour in ponds with and without fish in an urban landscape. The results show that urbanisation was not a determinant of water colour in the study ponds. The water colour gradient affected dytiscid beetles differently in the presence or absence of fish: In *ponds with fish*, dytiscid species richness and abundance significantly increased along the water colour gradient. In *ponds without fish*, water colour had no significant effects on dytiscid species richness and abundance. Water colour is an environmental determinant of dytiscid assemblages in urban ponds, and species responded to the water colour gradient differently.

### 4.1. Urbanisation Does Not Necessarily Drive Brownification in Urban Ponds

Urbanisation may affect the water colour of aquatic habitats, as anthropogenic activities have unignorable impacts on water chemistry in urban aquatic ecosystems [80]. In this study, the pond water colour exhibits a slightly decreasing but non-significant pattern along the urban gradient (Figure 1), which is in accordance with the negative correlation between water colour and the urban development in 51 Canadian lakes [7]. Many studies, however, show contradictory patterns compared to the findings of this study. For example, a study of 44 rivers in Sweden showed no correlation between urbanisation and total organic carbon concentration that is positively correlated with water colour [81]. In many countries, urban streams contain significantly higher DOC concentrations than rural and natural streams, possibly due to sewage leaks, landfill leaching, and wastes from industry and residential areas [6,32,33,35]. Such adverse impacts of urbanisation have also been found in groundwater at a global scale [82].

The non-significant correlation between water colour and urbanisation found in this study may have partially resulted from the strict environmental regulation and partially from the different extents of urban impacts on urban streams and ponds. In Finland, residential sludge cannot be discharged into waters, whether treated or not [83]. A similar regulation is practised in Sweden to mitigate eutrophication caused by nutrient release from anthropogenic structures [84,85], which was considered as an explanation for the lack of correlation between urbanisation and water colour in Swedish rivers [81].

Unlike urban streams and rivers, which are sometimes connected with stormwater pipes [86], urban ponds are often discrete habitats and not connected with other water bodies [87]. Some ponds in this study involve human disturbances, such as collecting mud and sediment soil [88] and leaf litter from the pond bottoms (Liao’s personal observations). Dissolved organic matter leaching from soil and leaf litter is a main source of natural water colour [89], a reduced amount of which is due to management practices for pleasant appearance [37] and may partially explain the slightly decreasing water colour along the urban gradient. In urban streams, urbanisation may lead to low organic matter retention due to increased scour [90,91], but organic matter input may increase when leaf fall increases from riparian trees [92]. In addition, the origins of organic matters may change due to urbanisation; headwater streams in New Zealand were found to experience a decrease of allochthonous organic matter but an increase in autochthonous dissolved organic matter (DOM) in urbanised areas, compared to streams in rural areas [93]. The finding in this study reveals that urbanisation itself may not necessarily be a driver of brownification in urban ponds, and future studies should investigate how coloured compounds and their origins affect water colour in urban lentic ecosystems.

### 4.2. Water Colour Has Complex Effects on Dytiscid Assemblages

Although the study ponds did not experience brownification, the responses of dytiscid assemblages to the water colour gradient shed light on the potential impacts of brownification on aquatic communities. In this study, the effects of water colour on dytiscids diverged in the presence or absence of fish. In *ponds with fish*, dytiscid species richness increased with increasing water colour (Figure 2a), whereas in *ponds without fish* there was no correlation between species richness and the water colour gradient (Figure 2b). This finding is inconsistent with the findings by Law and his colleagues that water colour had negative effects on water beetle species richness [49]. The positive correlation in *ponds with fish* indicates that brown water may provide prey refuge for dytiscids when fish are present, which is similar to the effects of aquatic vegetation on dytiscid species richness [53]. Similarly, zooplankton, such as copepods and cladocerans, also appear to benefit from highly coloured water [94,95], possibly because of the reduced feeding efficiency of some fish species, such as perch and sticklebacks [96,97]. 

Dytiscid abundance increased along the water colour gradient in both ponds with and without fish (Figure 2c,d), indicating that prey refuge is not the only benefit that highly coloured water can bring. Dytiscids are predators feeding on aquatic invertebrates and even vertebrates, such as tadpoles and fish larvae [55,59,98,99,100]. Brown water colour is known to have positive effects on the growth of tadpoles and the dry biomass of emergent frogs by enhancing nutrient availability in water [101] and benefitting mosquito larvae by eliminating UV light penetration [102]. The positive effects of water colour on potential prey imply that highly coloured water enhances food availability for dytiscids, the assemblages of which are partially driven by prey availability [103]. Dytiscids feed more efficiently when food density is high [104,105,106], which can be an explanation for the increasing dytiscid abundance along the water colour gradient in *ponds without fish*, despite species richness not changing. 

Although some dytiscids may benefit from highly coloured waters, there can be trade-offs between predator avoidance and foraging. *Ponds with fish* have increasing dytiscid species richness and abundance along the water colour gradient (Figure 2a,c) but still fewer species and individuals than *ponds without fish* (Table 1 and Table 2), indicating brown water mitigates but does not eliminate predation risk for dytiscids. Dytiscids, as prey, utilise visual, tactile, and chemical cues to detect predators [107,108,109,110,111]. When the visual cues are limited, dytiscids may alter their behaviour, such as reducing their activity, to avoid predators [109]. The reduced activity can negatively affect their foraging, especially when dytiscids need to search for prey actively under low food density [104,105,112]. In habitats with fish, the biomass of aquatic invertebrates is usually low [113,114], meaning that fish are not only predators but also competitors to dytiscids. Thus, in *ponds with fish*, even though brown water can provide some protection for dytiscids, there can be trade-offs between predator avoidance and foraging. 

### 4.3. Not Every Species Benefits from Brown Water

Although water colour appears to facilitate dytiscid abundance in both ponds with fish and ponds without fish, species respond to the water colour gradient differently. Some species, such as medium- to large-sized *Agabus* spp. and *Dytiscus* spp., appeared tolerant to highly coloured water, whereas some species, such as small-sized *Hyphydrus ovatus* and *Hygrotus* spp., tended to occur in clear waters (Figure 3a). Highly coloured water may negatively affect dytiscid antipredator defence. Small-sized dytiscids with colour patterns on elytra have similar mortality rates to medium-sized plain dytiscids in clear waters in the presence of dragonfly larva predators but higher mortality rates than medium-sized dytiscids in highly coloured water [115]. In this study, *Hygrotus inaequalis* and *H. impressopunctatus*, species with largely patterned elytra, were mainly recorded in clear water, possibly due to better environmental conditions for antipredator defence.

Highly coloured water may also decrease the feeding efficiency of small-sized dytiscids. For example, *Hyphydrus ovatus*, which was mainly recorded in clear waters, feeds on cladocerans and copepods [116]. Brown waters are known to provide zooplanktons, such as cladocerans, with prey refuges [94,117], which increases the difficulty of finding prey for water beetle species that feed on zooplanktons. Furthermore, some water beetles under other families, such as Haliplidae, are herbivores that often occur in clear waters [118] and feed on green algae [119,120,121,122], the growth of which can be strongly limited by highly coloured waters due to reduced light availability [12,14,123]; browning water may also negative affect such aquatic herbivorous invertebrates. Thus, highly coloured water may eliminate the occurrence of some water beetles by decreasing feeding efficiency and food availability.

One limitation of this study is that the water colour gradients in ponds with fish and ponds without fish have different ranges, with 10 to 220 mgPt/L in *ponds with fish* and 5 to 360 mgPt/L in *ponds without fish*, despite the standardisation of water colour before data analysis being able to improve the numerical optimisation process of GLMMs [67]. Aquatic invertebrates may respond to water colour differently in the presence or absence of predators [124,125,126,127]; the patterns obtained in the ponds without fish from the water colour range of 220–360 mgPt/L may not apply to the ponds with fish. Future studies on the effects of water colour on aquatic invertebrate communities should include a wider range of water colour gradients in both urban and non-urban landscapes, to investigate whether there are thresholds for different macroinvertebrate taxa, as found in zooplankton [128], and identify more general conclusions on the effects of water colour on aquatic biodiversity.

### 4.4. Implications for Urban Pond Management

Urban ponds provide many ecosystem services, such as supporting biodiversity and educational activities [129,130]. Water colour is one of the determinants of aquatic biodiversity [16,17,18,19,20] as well as people’s perceptions towards wetlands [37,131]. Understanding the effects of water colour on aquatic biodiversity is important in promoting the multifunctioning of urban ponds. In this study, urbanisation was not a determinant of water colour in urban ponds, yet the responses of dytiscid beetles to water colour shed light on aquatic biodiversity conservation in the browning aquatic world.

This study highlights the importance of considering interactions between brownification and other environmental factors, such as the level of predation risk and food density, instead of considering water colour as a solo factor. Browning waters may reduce the feeding efficiency of predators and provide prey species with refuges [96,97,117,132]. Yet, the conservation of specific taxa or species in browning waters should consider feeding relationships and trophic positions of the target organisms [133]. Aquatic invertebrates at higher trophic levels, such as dytiscids, may experience trade-offs between avoiding predators and preying on aquatic fauna. 

Some species are intolerant to highly coloured waters, which reveals that the increasing species richness and abundance along the water colour gradient in the presence of fish cannot be simply interpreted as brownification facilitating macroinvertebrates. Instead, the results found in the study highlight the importance of landscape heterogeneity. Urban habitat management could consider combining people’s perceptions and the biology and ecology of aquatic organisms to achieve multifunctioning of urban blue space. As people tend to appreciate clear water [37], based on this study, other environmental elements, such as aquatic emergent plants, can be added to urban ponds with clear water to meet the needs of aquatic organisms that prefer clear water and to better connect people with nature [134,135]; ponds with brownish water should be kept with more natural surroundings for conservation and educational values of different aquatic organisms and habitats.

## Figures and Tables

**Table 1:** Results of optimal GLMM with a Poisson distribution, modelling dytiscid species richness against the increasing water colour. The variance of sites as random effects is 1.13^2^, the variance of ponds as random effects is 0.70^2^; the variance of months as random effects is 0.17^2^. Estimated means estimated parameters. SE means standard error. ‘Fishless’ stands for ponds without fish; ‘ponds with fish’ was set as the reference level—the same occurs in Table 2.

	Estimate	SE	Z-Value	*p*-Value
Intercept	−1.76	0.51	−3.44	0.001
WaterColour	0.72	0.23	3.13	0.002
Fishless	1.34	0.53	2.53	0.011
WaterColour:Fishless	−0.76	0.25	−3.12	0.002

**Table 2:** Results of optimal GLMM with a negative binomial distribution, modelling dytiscid abundance against the increasing water colour. The variance of sites as random effects is 1.42^2^; the variance of ponds as random effects is 0.95^2^; the variance of months as random effects is 0.51^2^.

	Estimated	SE	Z-Value	*p*-Value
Intercept	−1.77	0.68	−2.60	0.009
WaterColour	0.76	0.16	4.72	<0.001
Fishless	1.94	0.67	2.92	0.003
WaterColour:Fishless	−0.83	0.16	−5.07	<0.001

## Data Availability

The data used in this study will be made available under reasonable requests.

## Supplementary Materials

The following supporting information can be downloaded at: https://www.mdpi.com/article/10.3390/insects15050308/s1, Figure S1: Map of the 26 study ponds at 11 sites in the Helsinki Metropolitan Area, Finland; Figure S2: Cross validation of the optimal Poisson GLMM modelling dytiscid species richness along the water colour gradient; Figure S3: Cross validation of the optimal negative binomial GLMM modelling dytiscid abundance along the water colour gradient; Table S1: Basic information about the study ponds; Figure S4: Stress of NMDS with different k; Figure S5: Simulated 1000 independent permutations of the original dytiscid assemblage dataset with different k; Figure S6: NMDS plots of dytiscid assemblages (A) along the urban gradient and (B) in the sampling months; Table S2: Poisson GLMMs modelling dytiscid species richness per trap; Table S3: GLMMs modelling dytiscid abundance per trap; Table S4: Results of optimal GLMM with a Poisson distribution, modelling dytiscid species richness against the increasing water colour; Table S5: Results of optimal GLMM with a negative binomial distribution, modelling dytiscid abundance against the increasing water colour.

## Appendix A

Full model description of Poisson GLMM of dytiscid species richness per trap:

$$Y_{ijk}\sim Poisson(\mu_{ijk}),$$

$$E\left(Y_{ijk}\right)=\mu_{ijk},$$

$$\log\left(\mu_{ijk}\right)=\alpha_{1}+\beta_{1}\times {WaterColour}_{ijk}+\beta_{2}\times {FishSituation}_{ijk}+\beta_{3}\times {WaterColour}_{ijk}\times {FishSituation}_{ijk}+a_{i}+b_{j}+\varepsilon_{k}$$

where dytiscid species richness in pond *i* at site *j* in the sampling round *k*, *Y_ijk_*, follows Poisson distribution with mean *µ_ijk_*. The term *µ_ijk_* is modelled in terms of water colour (*WaterColour_ijk_*), the presence or absence of fish (*FishSituation_ijk_*), and the interaction between water colour and the presence or absence of fish (*WaterColour_ijk_* × *FishSituation_ijk_*). The term *α*_1_ is the intercept. The terms *β*_1_, *β*_2_, and *β*_3_ are the parameters of covariates. The terms *a_i_*, *b_j_*, and *ε_k_* represent the ponds, the sites, and the sampling rounds as random effects.

The full model of dytiscid abundance per trap has the same description, except that *Y_ijk_* stands for dytiscid abundance and follows a negative binomial distribution.

## Appendix B

**Table A1:** Checklist of recorded dytiscids in the study ponds in Helsinki, Finland in 2021. The symbol ‘×’ stands for the presence of a species.

Genus	Specific Name	Ponds with Fish	Ponds without Fish
*Acilius*	*canaliculatus*	×	×
	*sulcatus*	×	×
*Agabus*	*affinis*		×
	*fuscipennis*		×
	*guttatus*		×
	*sturmii*	×	×
*Clemnius*	*decoratus*	×	×
*Colymbetes*	*paykulli*	×	×
	*striatus*	×	×
*Cybister*	*lateralimarginalis*	×	
*Dytiscus*	*circumcinctus*	×	×
	*marginalis*	×	×
*Graphoderus*	*cinereus*	×	
	*zonatus*	×	×
*Graptodytes*	*granularis*		×
*Hydroglyphus*	*pusillus*		×
*Hydroporus*	*angustatus*	×	×
	*erythrocephalus*		×
	*incognitus*	×	×
	*memnonius*		×
	*nigellus*		×
	*nigrita*		×
	*palustris*	×	×
	*striola*		×
	*umbrosus*		×
*Hyphydrus*	*ovatus*	×	×
*Hydaticus*	*seminiger*	×	×
	*transversalis*	×	
*Hygrotus*	*inaequalis*	×	×
	*impressopunctatus*		×
*Ilybius*	*angustior*		×
	*ater*	×	×
	*crassus*	×	×
	*fenestratus*	×	
	*fuliginosus*	×	×
	*guttiger*		×
	*quadriguttatus*	×	×
	*similis*	×	
	*subaeneus*	×	
*Laccophilus*	*minutus*		×
*Porhydrus*	*lineatus*		×
*Rhantus*	*exsoletus*	×	×
	*frontalis*	×	×
*Suphrodytes*	*dorsalis*		×
