# Supplementary material for: Water Colour Shapes Diving Beetle (Coleoptera: Dytiscidae) Assemblages in Urban Ponds
Source: Insects. 2024 Apr 25;15(5):308. doi: 10.3390/insects15050308 (PMC11122460; doi:10.3390/insects15050308)
Supplement: Supplementary file 1 [file insects-15-00308-s001.zip › insects-2963072-supplementary.pdf]

Supplemental materials

Water colour shapes diving beetle (Coleoptera: Dytiscidae) assemblages in urban ponds

Wenfei Liao[1,2,3,4*]

[1] School of Life Science, University of Electronic Science and Technology of China, No.4, Section 2, North Jianshe Road, 610054 Chengdu,Sichuan, China

[2] Ecosystems and Environment Research Programme, University of Helsinki, P.O. Box 65, FI-00014, Helsinki, Finland

[3] Department of Geosciences and Geography, Faculty of Science, University of Helsinki, P.O. Box 64, FI-00014, Helsinki, Finland

[4] Helsinki Institute of Urban and Regional Studies (Urbaria), FI-00100, Helsinki, Finland

Supplement 1

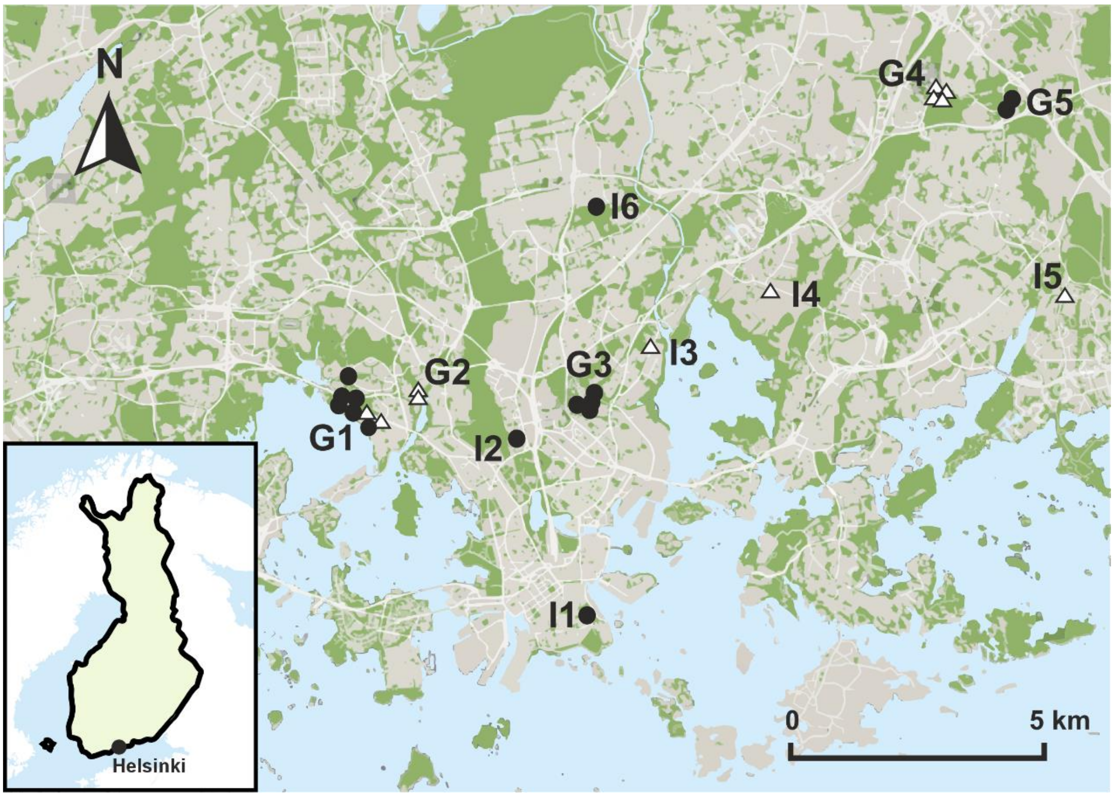

Figure S1 Map of the 26 study ponds at 11 sites in the Helsinki Metropolitan Area, Finland. The open triangles stand for ponds with fish, whereas the black circles stand for ponds without fish.

Table S1 Basic information about the study ponds. The pond ID is in accordance with Liao et al [87]. The pond water colour and pH show the mean values and the standard deviations. When there is only one value for the water colour and for the water pH, it means that the pond was sampled only once. The number of traps means the total number of traps set in a pond during the sampling month(s) in 2021. The dytiscid species richness and abundance show the total numbers of species and individuals obtained in a pond during the sampling month(s).

Pond ID	Fish situation	Water colour Mean ± SD (mgPt/L)	Water pH Mean ± SD	Nr of traps	Dytiscid species richness	Dytiscid abundance
I1	Fishless	10 ± 4	7.34 ± 0.28	15	1	1
I2	Fishless	220 ± 28	6.23 ± 0.31	19	8	102
I3	Fish	40 ± 15	7.05 ± 0.31	20	8	14
I4	Fish	33 ± 18	7.37 ± 0.01	16	2	2
I5	Fish	50 ± 18	7.12 ± 0.56	46	5	11
I6	Fishless	250 ± 99	6.27 ± 0.54	16	9	21
G1.1	Fishless	140	6.84	10	5	14
G1.2	Fishless	340 ± 28	6.76 ± 0.12	11	7	14
G1.3	Fishless	230 ± 127	6.84 ± 0.02	13	12	37
G1.4	Fishless	320	6.77	12	14	55
G1.5	Fishless	180 ± 85	6.85 ± 0.06	16	13	19
G1.6	Fish	143 ± 68	7.16 ± 0.33	55	15	108
G1.7	Fishless	200	6.52	6	11	16
G1.8	Fish	59 ± 23	7.25 ± 0.24	56	4	8
G2.1	Fish	63 ± 22	7.24 ± 0.97	38	18	80
G2.2	Fish	65 ± 35	7.49 ± 0.53	28	3	3
G3.1	Fishless	120 ± 45	7.74 ± 0.90	20	8	11
G3.2	Fishless	45 ± 13	7.66 ± 0.33	23	3	6
G3.3	Fishless	115 ± 90	7.04 ± 0.16	28	13	38
G3.4	Fishless	114 ± 88	7.10 ± 0.33	18	1	1
G4.1	Fish	24 ± 9	7.34 ± 0.10	32	0	0
G4.2	Fish	14 ± 3	7.30 ± 0.07	16	0	0
G4.3	Fish	30 ± 7	6.95 ± 0.14	40	0	0
G4.4	Fish	34 ± 13	6.80 ± 0.19	32	0	0
G5.1	Fishless	115 ± 21	6.44 ± 0.25	34	17	41
G5.2	Fishless	198 ± 77	6.54 ± 0.43	23	14	42

Supplement 2

Table S2 Poisson GLMMs modelling dytiscid species richness per trap.

Models	Description	AIC
**S1**	**~ WaterColour * FishSituation + (1|Month) + (1|Site/Pond)**	**1265.56**
S2	~ WaterColour + FishSituation + (1|Month) + (1|Site/Pond)	1273.34
S3	~ WaterColour + (1|Month) + (1|Site/Pond)	1276.76
S4	~ FishSituation + (1|Month) + (1|Site/Pond)	1272.25
S5	~ 1 + (1|Month) + (1|Site/Pond)	1288.92

Table S3 GLMMs modelling dytiscid abundance per trap, AP1 – AP5 modelled with a Poisson distribution; ANB1 – ANB5 modelled with a negative binomial distribution.

Models	Description	AIC
AP1	~ WaterColour * FishSituation + (1|Month) + (1|Site/Pond)	2176.91
AP2	~ WaterColour + FishSituation + (1|Month) + (1|Site/Pond)	2200.72
AP3	~ WaterColour + (1|Month) + (1|Site/Pond)	2205.57
AP4	~ FishSituation + (1|Month) + (1|Site/Pond)	2199.61
AP5	~ 1 + (1|Month) + (1|Site/Pond)	2205.37
**ANB1**	**~ WaterColour * FishSituation + (1|Month) + (1|Site/Pond)**	**1318.19**
ANB2	~ WaterColour + FishSituation + (1|Month) + (1|Site/Pond)	1320.67
ANB3	~ WaterColour + (1|Month) + (1|Site/Pond)	1320.12
ANB4	~ FishSituation + (1|Month) + (1|Site/Pond)	1328.74
ANB5	~ 1 + (1|Month) + (1|Site/Pond)	1336.77

I simulated 10,000 datasets with the optimal models to ensure that the optimal models were able to cope with the high percentage of zeros in the dataset of dytiscid species richness and abundance and it was unnecessary to run zero-inflated models [67].

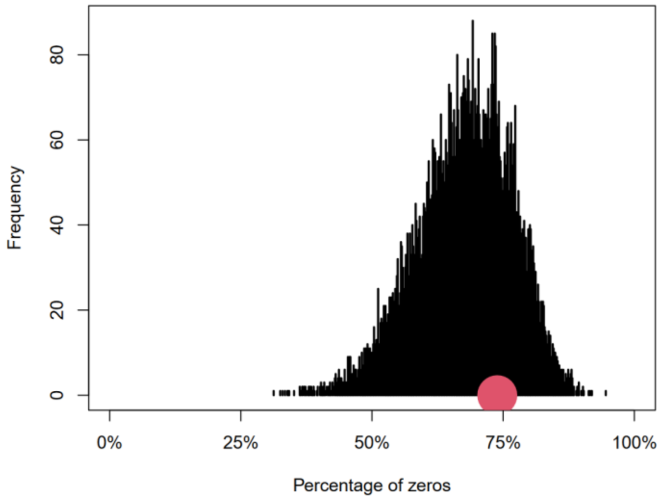

Figure S2 Cross validation of the optimal Poisson GLMM modelling dytiscid species richness along the water colour gradient. The black bars stand for the frequency of the datasets that contain certain number of zeros simulated from the optimal Poisson model. The pink dot stands for the percent of zeros in the dytiscid species richness dataset collected in 2021 for this paper. The same below.

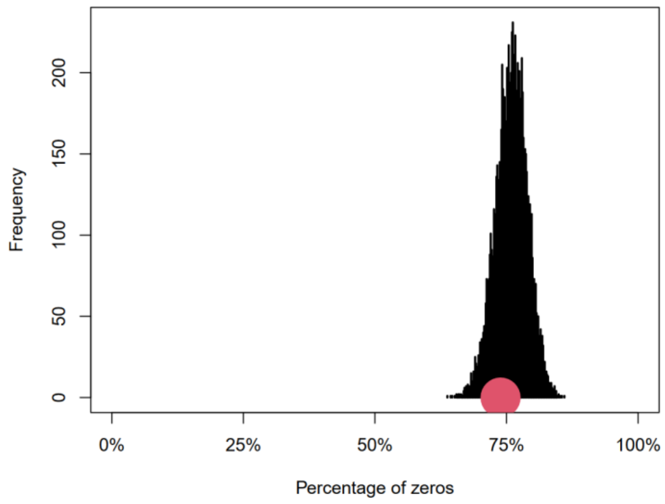

Figure S3 Cross validation of the optimal negative binomial GLMM modelling dytiscid abundance along the water colour gradient. The pink dot stands for the percent of zeros in the dytiscid abundance dataset.

Supplement 3

Results of GLMMs with 'ponds without fish' set as the reference level.

Table S4 Results of optimal GLMM with a Poisson distribution, modelling dytiscid species richness against the increasing water colour. Ponds as random effects is 1.12^2; months as random effects is 0.17^2. Estimated means estimated parameters. SE means standard error. 'Fish' stands for ponds with fish; 'ponds without fish' was set as the reference level.

	Estimate	SE	Z-value	p-value
Intercept	-0.42	0.48	-0.87	0.386
Water Colour	-0.04	0.11	-0.40	0.693
Fish	-1.34	0.53	-2.53	0.011
Water Colour : Fish	0.76	0.25	3.12	0.002

Table S5 Results of optimal GLMM with a negative binomial distribution, modelling dytiscid abundance against the increasing water colour. Ponds as random effects is 1.09^2. 'Ponds without fish' was set as the reference level.

	Estimated	SE	Z-value	p-value
Intercept	0.17	0.65	0.27	0.791
Water Colour	-0.07	0.10	-0.74	0.458
Fish	-1.94	0.67	-2.92	**0.004**
Water Colour: Fish	0.83	0.16	5.07	**<0.001**

Supplement 4

Model validation of NMDS, selecting the smallest number for k.

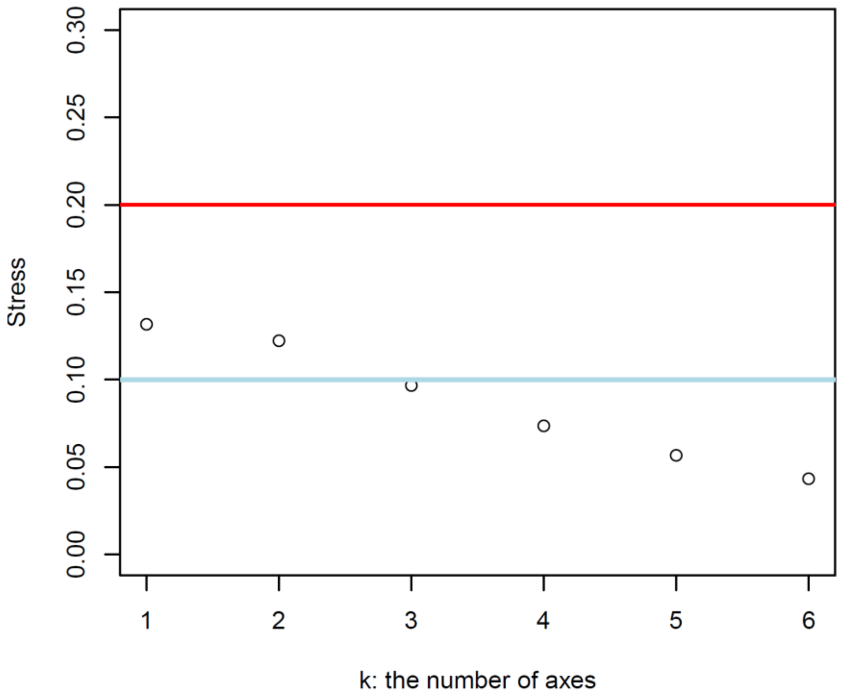

Figure S4 Stress of NMDS with different k.

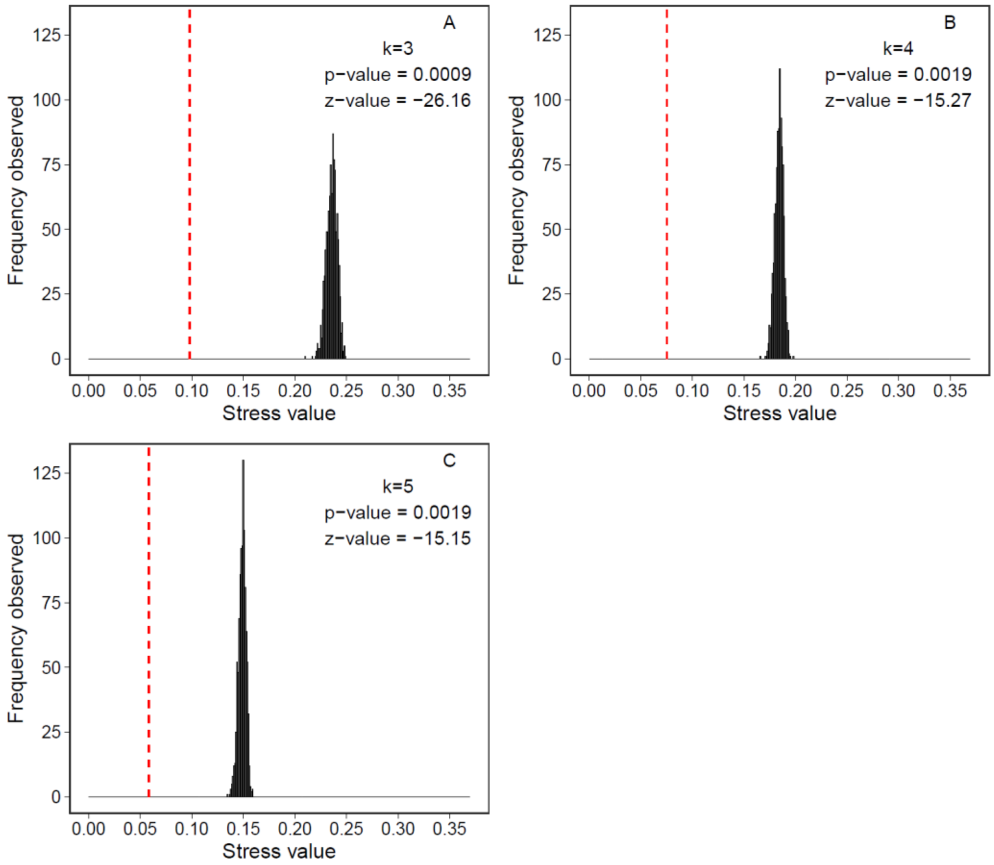

Figure S5 Simulated 1000 independent permutations of the original dytiscid assemblage dataset with different k.

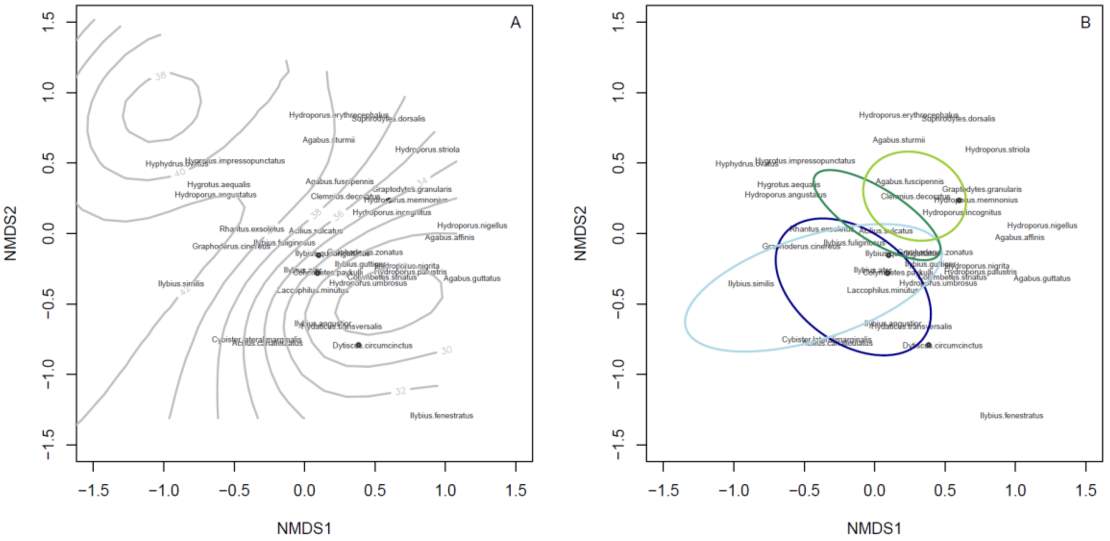

Figure S6 NMDS plots of dytiscid assemblages (A) along the urban gradient and (B) in the sampling months.
